# How to Control Behavioral Studies for Rodents—Don’t Project Human Thoughts onto Them

**DOI:** 10.1523/ENEURO.0456-20.2021

**Published:** 2021-01-25

**Authors:** Lisa Genzel

**Affiliations:** Donders Centre for Cognition and Behaviour, Radboud University, 6525 AJ, Nijmegen, The Netherlands

**Keywords:** behavior, memory, mice, rats

## Abstract

In neuroscience research, we often use behavior as an easy tool and assume a straightforward relationship between memory and behavior. However, many factors are often not accounted for and need to be considered when interpreting a behavioral outcome. This opinion article will discuss factors in rodent studies such as handling and how the animal views the world, that will affect whether memory leads to a certain behavior.

## Significance Statement

Memory and behavior do not have a direct relationship, instead many other factors will influence whether memory leads to a certain behavior. Here I discuss some factors such as what the animal attends to within a task, how motivated an animal is, or what its inner state is. Further, the article suggests some ideas, such as better handling and habituation practices, that we can implement to deal with these issues.

## Introduction

**Figure 1.**
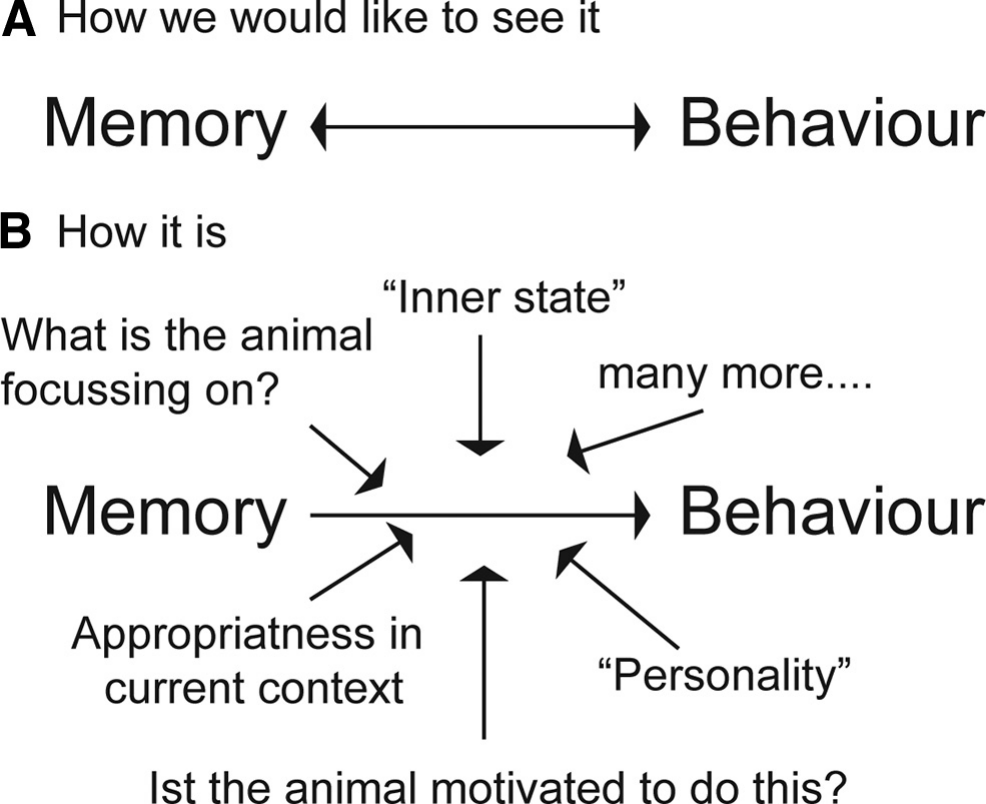
***A***, Many researchers think of behavior as a direct readout from, for example, memory and believe that since the memory has to be present for a certain behavior, the reverse logic is also true: if the behavior is not present, the memory must not be present as well. ***B***, But actually, one can only draw the logical conclusion that memory may lead to a certain behavior if the behavior expressed is influenced by many other factors as well.

In neuroscience, we like to test for a certain behavior and then use that as direct outcome to determine underlying mechanisms. This can make the forced swim test, tail suspension test, or open-field exploration to conclude about a depressed or anxiety state of the animal or any form of memory task to test whether a memory is intact or not. Since memory needs to be present to be able to observe the learned behavior, the tendency is to use the reverse inference: if the behavior is missing, we can conclude that the memory is not intact (Fig. 1*A*, how we like to see it). However, in the case of behavior the directionality of evidence is a one-way street and cannot simply be reversed. It is not behavior = memory. Instead, many other factors will influence whether the presence of memory (or depressed mood) leads to a certain behavior. To name just a few of the factors, what the animal is focusing on, how the animal views the world, and the appropriateness of the behavior in the current context will influence behavior (Fig. 1*B*, how it is). Therefore, if the behavioral expression of memory is not present, it does not mean memory is not intact.

One should keep in mind, with a few exceptions, that most such influencing factors have not been systematically tested for. Thus, much of what is described in this opinion piece is based on personal experience or anecdotes shared among behavioral researchers.

## What is the animal focusing on?

Is the animal focused on the task and doing what you think it is doing? Of course, this is closely associated with the next point: is the animal motivated to perform the behavior that you want it to perform? Recent studies have shown that, what many have categorized as “noise” in neural recordings is actually associated with the animal doing other things than the intended task, such as subtle facial movements (Stringer et al., 2019). This is especially important when we use tasks that have just one trial as a test. If the animal is distracted and not focused on the task, you may not see the expected behavioral outcome. Distraction can be both internally generated (e.g., being more interested in grooming) and externally originated (e.g., changes in housing or testing environment can distract an animal). We might think that we can control for the external distracting factors; however, we need to keep in mind that animals will sense things we cannot. For example, their sense of smell is superior to ours and since their hearing range is different, they may hear sounds we do not hear. Very high-frequency or low-frequency sounds can be generated, for example, when drilling into foundations far away. Interestingly, the more one tries to control for such distractors by exclusion, the more salient and distracting they are to an animal when encountered. In contrast, if an animal is used to a very noisy environment, it seems to deal better with such distractors and is able to ignore them.

## Motivation, “personality” and the “inner state”

How motivated an animal is to do something will also influence if memory leads to a certain behavior. Motivation is not only affected by known factors such as introducing food deprivation to create motivation in an animal to look for food in a maze. Also the individual personality (for lack of a better term) of an animal will affect its general motivation. Most experimenters working with behavior will report certain types of personalities in rodents, especially noticeable in rats but also present in mice: across eight animals, one usually has one to two animals who never seem very motivated to do anything; these tend to be larger, heavier animals. There will also be at least one hyperactive animal that has issues focusing on the task at hand. These differences are usually discussed as anecdotes, but the regularity of the occurrence of different types within a cage of four animals does indicate that it might be a result of hierarchies in the cage and may represent a natural way of creating diversity in a population. Since one of four animals will usually have such a noticeable personality, which will have a large influence on motivation and consequently expressed behavior, one should be careful when interpreting individual memory expression. For example, the larger animal may not express the behavior associated with a memory because it is not motivated to pay attention to the task, but that does not mean the memory is not present in that animal.

Further, manipulating motivation does not always follow a simple linear relationship (Berditchevskaia et al., 2016). One good example is food deprivation: some food deprivation will lead to increased focus when searching for food and can motivate an animal to deliberate before deciding where to search for food. However, too much food deprivation will lead to faster search strategies that tend to include less deliberation. This U curve in food deprivation, with too little or too much causing worse expression of deliberation and subsequently memory performance, might be especially seen in tasks that are cognitively more demanding. An animal that is too food deprived tends to “think” less.

The inner state of an animal will also influence how it will behave in a task. The way any type of procedure will influence the inner state of the animal can show a natural variability across animals. A great example of the diversity of possible outcomes was shown by Fogel et al. (2011). All animals underwent the same avoidance-learning paradigm (footshock in the dark part of the box). However, later on some remembered and became active avoiders (i.e., the next day they avoided the shock zone). A second group seemed to “forget” and returned to the part of the box with the shock the next day, but as soon as the shock was reinstated, they left the zone again. Interestingly, a third group showed evidence for learned helplessness: they would go to the zone the next day and, even if shocked again, remain there. Thus, the same procedure resulted in some cases in an inner state of “depressed-like mood” or “learned helplessness,” which influenced the behavioral response. It also remains unclear whether those animals “remembered” the initial experience or not. Similarly, a “depressed” animal would be less likely to be motivated to run for a food reward if it is feeling anhedonia. Thus, the inner state would influence performance in reward-related tasks independently whether an animal remembered learning about the reward or not.

Another example for intrinsic factors influencing results can be seen in the object exploration task. Generally, we use the discrimination index as an outcome in this task, for which we calculate the difference in exploration between the known and the new object. This index is based on the preference for novelty—neophilia—with animals on average exploring the novel object more than the known one. However, the difference in genetics, disease, or other factors that one plans to test in an experiment can potentially change the preference from general neophilia to neophobia. Subsequently, this would lead to a change from a positive discrimination index to a negative one, if the animal remembers the original event. Moreover, a mix of neophilia and neophobia will result in a discrimination index of zero even if the memory is present. This is important to consider, since the natural tendency of rats in the wild is neophobia. Only with domesticated rats do we see a natural tendency of neophilia.

## Appropriateness of behaviors in the current context

Another, often neglected, factor is the appropriateness of a behavior in a certain context. In tasks involving fear-conditioned responses, this is of special importance. Many memory researchers use freezing to detect the presence of memory. However, if freezing is the appropriate response to fear, it is very much dependent on the context. First, if freezing versus escaping is the correct response to fear, it is dependent on the possibility of escape (e.g., size of the box) as well as on the distance to the fear-inducing cue (Fanselow, 2018). Second, an animal may remember the fear-inducing event, but “realize” it was a one-time event and will not happen again. As a result, the animal should not show the freeze response despite the fact that the memory is intact. Or at the other extreme, if an animal is never handled by an experimenter and the only event it ever experienced was the fear-conditioning paradigm, it would be more likely to generalize the fear to any handling procedure by the experimenter and the fear response will be less specific. In sum, how an animal will respond to a fearful paradigm is mainly influenced by habituation procedures occurring before and after the memory-inducing event (Wang et al., 2009). This also means that the presence of freezing, specific or generalized, is very hard to directly associate with the presence or absence of a memory. As a point in case: the same principle of fear generalization from the original conditioning box to another box is used by memory researchers to discuss systems consolidation from the hippocampus to the cortex and by disease researchers to discuss the development of post-traumatic stress disorder after a trauma event.

The expression of appetitive behaviors is similar: an animal will not eat in an unfamiliar or changed environment. It needs to feel safe and to ensure the absence of danger before it will go to another type of behavior such as food consumption. Consequently, how familiar an animal is with the test environment will influence food search behavior and consumption.

## Other influencing factors

So far, a few of the factors that can influence behavior have been mentioned, but, of course, there are many more. For example, the time of day at which training and testing is performed can have a large influence on both memory and behavior. Circadian oscillations can be seen in gene expression and kinase activity (Eckel-Mahan et al., 2008), and diseases can additionally affect these circadian oscillations; consequently, the time of day can differentially affect results in wild-type and disease models (Debski et al., 2020). Also, how animals are housed—individually or in groups—can affect disease expression (Bernard, 2019) as well as behavior. For example, in group-housed animals fear responses can be socially transmitted to nonshocked cage mates (Carew et al., 2018). Furthermore, animal-handling techniques such as cupping versus tail pickups can have a large effect on behavior (Hurst and West, 2010; Gouveia and Hurst, 2017), as can the sex of the experimenter (Sorge et al., 2014). It has been shown that male experimenters create an initial stress response in rodents that can affect experimental outcomes.

## Rodents do not see the world as we do

One larger issue in rodent behavior, especially rodent learning tasks, is our preference to set up tasks using our human way of thinking and sensory input. Tasks tend to be based on visual input and cues, despite the fact that rodents are not as visually oriented as we are. The active time of rodents in nature is dusk and dawn, when not much light is present. Further, their natural home environments are complex underground burrow systems, which will not have a light source. This is why they rely more on olfactory cues as well as their sense of touch via whiskers. Rats also can use their anal glands to mark food areas as well as danger zones with specific smell cues.

**Figure 2.**
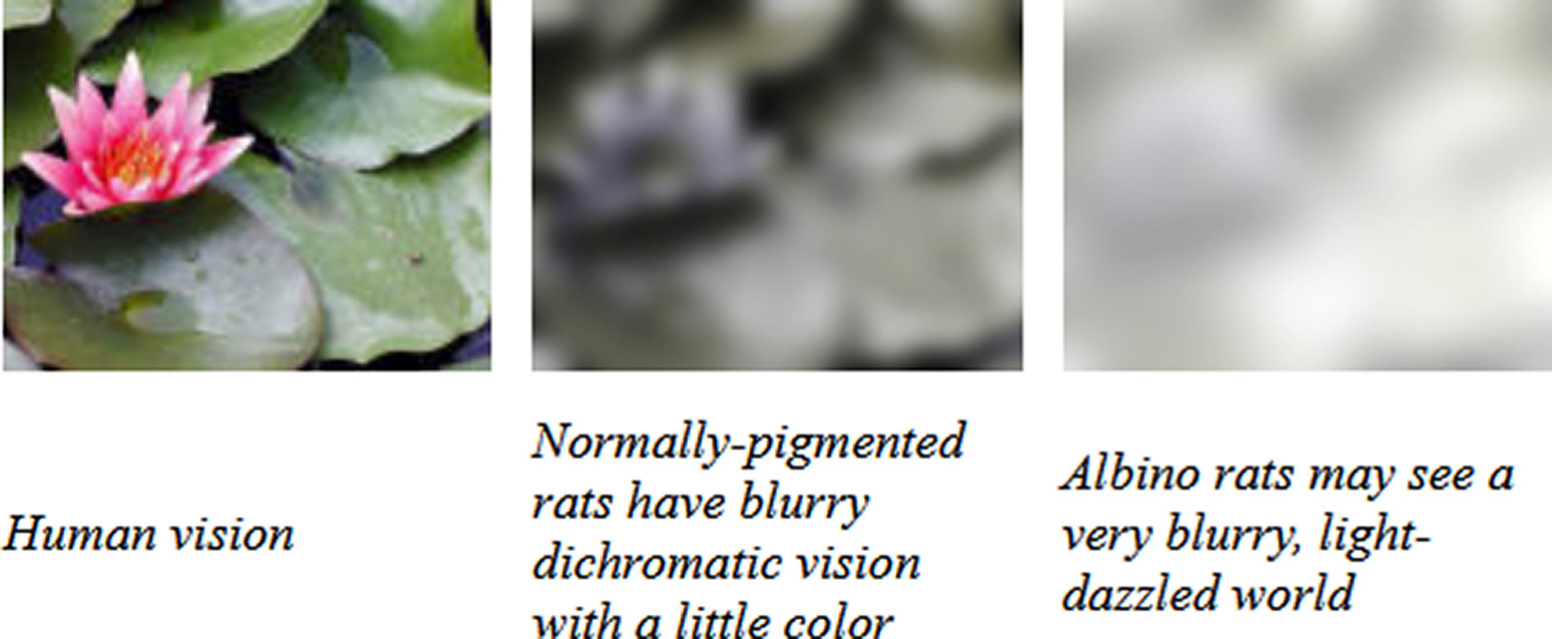
How rats see the world. Rats (and mice) are very short-sighted and do not have the receptors for the color red. While they have the receptors for other colors, they do not naturally pay attention to them. Thus, what they see is very different from what we see, which will affect how they will perceive certain visual cues used in experiments. Top, Left to right, what a human would see, a pigmented rat (e.g., Lister Hooded or Long–Evans strains) would see and what an albino rat would see. Taken from http://www.ratbehavior.org/RatVision.htm.

Many researchers do not consider that rats perceive red as black (since they do not have the red receptors), and while they can see color they need to be trained to perceive and process colors. Further, they are short sighted and, in the case of albino rats, would even in human terms count as legally blind (http://www.ratbehavior.org/RatVision.htm). For examples of rat view, see Figure 2 and http://www.ratbehavior.org/RatCam.htm. Thus, what we humans may perceive as good cues and experimental setups will be viewed very differently from the rodent perspective.

The most extreme example of using human perception to design rodent experiments is the extensive use of virtual reality that is only based on visual input. The dissociation between any potential olfactory or tactile cues on the ball or treadmill used will make it even more difficult for the rodent. Most likely many virtual reality tasks are not measuring what they think they are. For example, Aghajan et al. (2015) showed that virtual reality setups that do not consider olfaction or tactile stimuli are more likely measuring distance estimation of the animal than spatial perception.

## Which strategy is the rodent using?

One consequence of relying too much on our perception of what we think the animal should be doing is the rodent using a different strategy to solve a task than the one intended by the experimenter. A classic example would be finding a hidden platform in the water maze. While we like to think that an animal will use an allocentric strategy to learn the place of the platform within the environment, placement of cues, or even the sex of the animal, can result in the animal using a different strategy that relies on other brain areas. For example, having the platform close to a large dominant cue (e.g., the bunched up curtain hanging next to the maze) could lead to the animal using a beaconing strategy in which the animal just homes in on that one large beacon instead of using an integrated spatial representation of the environment. This strategy would be independent of the hippocampus in contrast to the allocentric spatial strategy. Another common strategy used by some animals is, instead of learning the location of the platform, learning the distance to the wall. If the animal always swims in a circle around the maze with a certain distance to the wall, it will find the platform more reliably than by using a spatial strategy.

## Rat and mouse behavior

One key concept to keep in mind is that a rat is not a little human. Each species is specialized for certain behavior and will express it in their own way. We humans are very good at rule learning, but this is not the most natural form of learning for rodents. This is why it can take them quite a long time to learn tasks based on rules such as seemingly simple alternation or win–stay tasks, but can learn to navigate flexibly to a goal location, planning direct paths in a 9 × 5 m maze within 20 min of first exposure (unpublished data from the HexMaze in the Genzel laboratory; https://www.genzellab.com/#/hexmaze2/).

Also, a mouse is not just a little rat. Rats and mice differ in their baseline behavior. In a large maze environment, mice show much less goal-oriented behavior than rats (unpublished data from the HexMaze in the Genzel laboratory; https://www.genzellab.com/#/hexmaze2/). Further, Jones et al. (2017) could show that rats and mice differed in levels of baseline activity measured as shuttle rate during intertrial intervals; mice shuttled two to three times as frequently as rats. Species differences in behavioral ecology may underlie this difference, as mice need to move rapidly when outside of burrows to minimize predation risk. They tend to use bursts of speed to run from a more sheltered position to the next. Thus, when designing a behavioral experiment, we should not aim to test rats and mice in cognitive skills derived from human abilities; instead, we should test them on behaviors that fit their ecological niche.

Recently, Gomez-Marin and Ghazanfar (2019) extensively and beautifully discussed that it is impossible to treat animals as passive stimulus–response machines. Instead, we must consider their behavior in the context of their species-specific bodies and natural environment. A rat is not a little human, a mouse is not a little rat, and flies are not cheap mice. In addition, the history of an individual will influence their behavior. Variability is built into biological systems to increase possible adaptability to changes, and thus even inbred mice raised under identical conditions will show substantial variability.

Further, we tend to think of behavior as output, which is intended as our input. Instead, for the organism, what it does is much less important than the consequence of what it does. Thus, the behavioral output of an organism is aimed at influencing the input the organism receives, and we should move to this organism-centric view. For a more detailed discussion of this and how the three biological principles of materiality, agency, and historicity, as well as the idea of “Umwelts” should be considered in the study of animal behavior, please see the studies by Gomez-Marin (2019) and Gomez-Marin and Ghazanfar (2019).

A separate issue is reading human cognition into certain animal behaviors, such as “regret” when an animal is just looking to a past location. One should stick to describing the observed behavior (e.g., looking back) without reading any human emotional value into it, even if it may be tempting to do so to achieve higher impact and press mentions. Buzsáki (2020) nicely summarized these issues recently in another opinion article. In this article, I sometimes used such terms as personality, mainly for lack of better terms.

## Differences can cause variability across laboratories

The International Brain Laboratory has mounted an impressive effort to create standardized behavioral tests and datasets across different laboratories. Their recent preprint highlights how variable rodent behavioral performance can be across laboratories. Mice were trained in a decision-making task in seven different laboratories, and, once training was complete, the mice performed very similarly across the different laboratories (Aguillon-Rodriguez et al., 2020). However, how long it took to complete training was very variable and ranged from 3 to 59 d. This huge variability is likely to be the result of exactly those factors described above, which causes the variability between mice within each laboratory (e.g., personality) as well as group variability between laboratories (e.g., how animals are routinely handled in each laboratory). In their impressive efforts to standardize, the International Brain Laboratory describes habituation practices to the apparatus but not how the animals are handled both before habituation as well as during training. Perhaps including standardization in those practices as well as considering the sex (Sorge et al., 2014) and consistency of the experimenter (is it always the same person or do different people train the animals?) could help reduce the variability during initial training.

## What should we do?

How should we deal with these influencing factors? We want to use behavior as an outcome to be able to deduct what happened to the memory. The most important consideration is to be aware of the potential influencing factors and confounds. Only then can we even try to control for them.

For example, it is important to look at the distribution of behavior across the individuals. In the neophobia/neophilia case during object exploration, looking at clusters of animals across the positive and negative discrimination index can be helpful. Is there a normal distribution or do subclusters seem to be present?

Habituation procedures and everything that happens to the animal outside of the experimental task is also critical. These will influence what the appropriate behavior is in the context as well as the inner state of the animal. For example, how well an animal is handled and its intrinsic fear and apprehension toward the experimenter as well as general procedures will influence the outcome of any task (Hurst and West, 2010; Gouveia and Hurst, 2017). Currently, it is not common to describe general handling procedures that occur before training beyond the very short description of “animals were handled for 10 min a day before the experiment.” A potentially better way to describe and therefore to standardize such procedures could be the use of videos (see https://www.genzellab.com/#/animal-handling/). It is important to include details of the habituation in the experimental description. Were animals habituated to handling with active techniques that simulate rough tumble and play? Or did the experimenter place the hand in the cage and let the animals approach? We have tested both types of handling in the laboratory. Interestingly, while active tumble and play led to less apprehensive animals that voluntarily approached the experimenter, the other approach (only putting the hand into the cage) led to more apprehensive animals and to biting the hand of the experimenter. Further, how was the animal taken from the cage? By cupping or tubing techniques or with tail pickups? How often were the animals handled outside of the experimental context? How often were they brought to the experimental room before training or testing was performed? More handling and habituation as well as nonaversive pickups will lead to less apprehensive animals and, better, to more consistent behavioral results.

Handling should also be considered before numbering/labeling of animals and group allocation are performed. The natural personality (see above) of an animal will lead to an animal being more likely to be in the front of the cage when the experimenters first approach. As a result, if the numbering of the animals within a cage is done before any handling, the same types of animals will consistently have the same animal number. Instead, one should do the numbering of animals at a random, later time point during initial handling. For example, 10 min into the third handling day when all animals are used to the experimenter.

Experimenters need to be aware of general procedures in the animal unit as well as in the building to reduce distractors. Especially on critical days, such as a test day, one should avoid any changes or other novel experiences for the animal. This could be a new home cage because of cage cleaning or an unfamiliar person entering the housing room for maintenance or renovation two floors away in the same building. All of these factors can lead to more generally alert, less focused animals.

We also need to describe more details of the experimenter. Was it one constant experimenter throughout the whole period or were different experimenters involved? How well trained was the experimenter? Was it a new intern or an experienced animal trainer? What was the sex of the experimenter? Since male experimenters will cause a stress response in rodents independent of how familiar the animals are with the person, these details are critical. In our experiments, we ensure that the animals are brought to the experimental room and left for at least 20 min after transport before any behavior is performed. Like this, the animals can calm down after the transport experience as well as accommodate to the training room and current experimenter. Further, while multiple experimenters will train the same animals and they may be less experienced, each experimenter has “met” and handled each animal at least once before in the housing room outside the experimental context.

We should aim to use behavioral tests that fit the ecological niche of the animal. When designing an experiment, one should consider what the behavior could correspond to in the wild. Further, tasks involving fear should only be used when the research question pertains to that system. Fear memories are not a good model for “simple,” declarative memory.

One approach that can help when determining the daily experimental schedule is trying to see things from the point of view of the animal, especially when it concerns the sequence of experiences the animal will undergo. As an example, we can take an appetitive task with a food cue association, during which each trial starts with a food cue that should then lead to a correct choice. If after two wrong choices the animal is “punished” by stopping the current trial and then immediately starting the next one, the animal may not perceive it as punishment since each trial starts with eating the cue pellet. Instead, it will learn that if it makes two random choices, it will be picked up and receive a reward. From the human viewpoint, it seems like a punishment; from the rodent viewpoint, random behavior is the easier way to receive a treat.

One should also consider the animal point of view when choosing the visual cues in spatial tasks. One helpful trick is filming the task environment at the height of the rodents and implementing potential rodent movement patterns such as looking up or rearing. These videos (or pictures) can then be rendered to rodent view, using techniques mentioned in http://www.ratbehavior.org/RatVision.htm. However, even without this rendering, simply viewing the layout and selection of cues from the perspective of the animal can be very enlightening for the experimenter. We generally recommend using high-contrast cues that are arranged asymmetrically (e.g., when implementing in a small box environment). Further, 3D cues are much more useful to the animal than 2D cues, especially when two 3D cues are arranged to have unique overlap patterns when viewed from different places in the testing area. To compensate for the short-sightedness of the animal, there is a simple rule of thumb: when viewing the cue with one eye closed from the height of the animal, it has to be wider than two fingers of the experimenter’s hand (with the arm stretched out). Then it is large enough for the animal to see.

Finally, we should value the expertise of behavioralists. An experimenter with experience in behavior can determine, just by watching an animal, whether it is too hungry, not focused, or distracted by an external stimulus. We readily accept that it takes experience to be able to run sophisticated techniques such as optogenetics, electrophysiology, or calcium imaging, but expertise in behavior is often less valued. Many laboratories believe that they can take any behavioral paradigm and apply it without consulting experts. This is not the case.

## Conclusion

In sum, behavior is not a simple tool or measurement such as measuring the activity of individual neurons or a brain area. Instead, we should always keep in mind that behavior is the outcome of many different factors. Thus, for one, we should be aware of these factors, keep track of them, and, ideally, control for them as best as we can. Currently, most such influencing factors have not been systematically tested for, with a few exceptions (Hurst and West, 2010; Sorge et al., 2014; Gouveia and Hurst, 2017). Therefore, much of what is described in this opinion piece is based on personal experience or anecdotes shared among behavioral researchers, highlighting that more systematic research on this topic is needed.

All of the additional measures we take to control for these issues, such as habituation to the experimenter and time of day of the experiment, need to be described in more detail in scientific articles than is currently common practice. The current practice of leaving out such details is most likely one of the reasons that many behavior protocols work in one laboratory but not necessarily in other laboratories. Further, we should consider moving to paradigms that are more natural and significant for each species. Finally, when coming to conclusions on the results of studies including animal behavior, we need to keep in mind that the absence of a certain behavior is not conclusive evidence on the absence of, for example, a memory trace.

## Synthesis

Reviewing Editor: Macià Buades-Rotger, University of Luebeck Decisions are customarily a result of the Reviewing Editor and the peer reviewers coming together and discussing their recommendations until a consensus is reached. When revisions are invited, a fact-based synthesis statement explaining their decision and outlining what is needed to prepare a revision will be listed below. The following reviewer(s) agreed to reveal their identity: Alejandro Gómez-Marín.

Dear author,

Thanks for your invited opinion article on the operationalization of cognitive processes in rodent studies. Though difficult to address, this is an important topic because researchers often assume simple relationships between particular behaviors and underlying functions like memory. Though this topic is not completely ignored, and many researchers design studies with these limitations in mind, this article is still valuable because it will bring attention to these issues, which are critically important. While straightforward and succinct, the manuscript could benefit from some more precision in some parts. Here is a synthesis of the reviews:

- Significance statement: the author should elaborate more on the paper’s key contributions, so that readers can grasp not only what the article is about but also what its central points are.

- In the abstract, it would be useful to mention that the article focuses on rodent studies.

- In the Introduction it is stated that the remainder of the article will focus on the memory example, but this doesn’t appear to be the case. This sentence could be removed or reformulated to acknowledge that the author focuses mostly, but not exclusively, on memory.

- Many anecdotal statements are made throughout the article with no references to back them up or explain them further. This would be most useful for scientists who do not work with rodents and may be quite unfamiliar with some of these issues. Just a few examples among many others include lines 38-39, lines 45-56, lines 60-64, and others. If there are no appropriate references for some of these statements, it would be useful to provide some data from the author’s own experiences, perhaps a figure showing real examples of the behaviors being described. This would expand the usefulness of the article.

- Some of the subheadings in the article are confusing or vague. For example, what is meant by "inner state", and how does that differ from personality and motivation? "Habituation and awareness" is also an unclear subheading, and the paragraph following this subheading does not clearly relate. The author should either clarify the criteria for this classification, or perhaps restructure the headings according to more standard distinctions (e.g. awareness can be lumped together with attention, and the "inner state" section can be considered as tapping into motivation).

- The article sets up and describes a serious issue in neuroscience, but falls short of providing suggestions on how things can be improved. One suggestion is to "trying to see things from the animal point of view, especially when it concerns the sequence of experiences the animal will undergo". But this is vague, and is not easy for researchers to implement. Although thoroughly addressing these issues is perhaps beyond the scope of the article, a suggested protocol or clear list of suggestions to follow would be helpful.

- Some of the claims have appeared in Gomez-Marin & Ghazanfar 2019, perhaps the paper can use it to strengthen and/or complement their claims?

- Given the recent impressive efforts form the International Brain Lab, it would be worth discussing specifically the extent to which the author’s points are being taken care in the sophisticated approach of the IBL. I suggest that the author writes a new subsection (or perhaps a text box) analyzing the procedure of the recent bioRxiv paper of the IBL.

- It would be interesting and helpful to provide a figure or schematic to illustrate how many different behaviors have been linked to different mechanisms across different studies, though this is not required.

- Lines 162-163: "Most likely many virtual reality tasks are not measuring what they think they are." What are they then measuring? Perhaps the authors can briefly elaborate on this?

- Perhaps the paper could benefit from introducing the concept of Umwelt as described by Von Uexkull, which has become a central notion in zoology, but is largely ignored in neuroscience. The author can also take a look at this recent piece "A clash of Umwelts: Anthropomorphism in behavioral neuroscience". On a related note, the author could consider discussing the difference between anthropomorphism and anthropocentrism.

- It is interesting that the author mentions Buzsaki at the end of the piece, while still using psychological categories such as "attention" or "motivation" (see figure 1). This is somewhat contradictory. Please clarify the position.

- Finally, a more generic but potentially crucial issue concerning the author’s argument: after the welcome and necessary insistence on being animal-centric, the author still conceives behavior as "output", rather than the control of input. Namely, for an organism, it is much less important what it DOES than the consequences of what it does, which are always somewhat necessarily assessed through its perception. This "closed-loop" view of animal behavior is, in one reviewer’s opinion, truly organism-centric, while thinking of behavior as "output" ("open-loop" view) betrays another kind of anthropomorphism since the output is what we humans can measure. In other words, the output of the organism is our input, while we should strive to think of their output as a means to control their input. Can the authors speak to this point (maybe in the context of the Gomez-Marin & Ghazanfar paper mentioned above)?

- There are a number of grammatical errors that make the article difficult to read in some places.
